# Editorial: Understanding Startups: From Idea to Market

**DOI:** 10.3389/fpsyg.2022.876172

**Published:** 2022-03-22

**Authors:** Yenchun Jim Wu, Chih-Hung Yuan, Mu-Yen Chen

**Affiliations:** ^1^College of Humanities and Arts, National Taipei University of Education, Taipei, Taiwan; ^2^Graduate Institute of Global Business and Strategy, National Taiwan Normal University, Taipei, Taiwan; ^3^School of Economics and Commerce, University of Electronic Science and Technology of China, Zhongshan Institute, Zhongshan, China; ^4^Department of Engineering Science, National Cheng Kung University, Tainan, Taiwan

**Keywords:** entrepreneurship, talent cultivation, start-up, business venture, entrepreneurship education

With the onset of the COVID-19 pandemic, the market has become increasingly unpredictable. Therefore, new opportunities and threats for organizations emerge, forcing the adaption of capabilities to the changing environment. In this environment, the ability to promptly bring ideas to the market has become unprecedentedly important. Focusing on the interaction between organizations, entrepreneurship, education, and talent cultivation, 61 academic papers from 207 authors were reviewed in this research topic. The following sections will elaborate on organizations, new entrepreneurs, education and talent cultivation, respectively.

## Organizations

From the social norm perspective, Du et al. explored how norms affect individual creativity and how such creativity subsequently affects leadership. They found that social norms effectively promoted individual creativity, which consequently had a positive impact on transactional and transformational leadership. Furthermore, they determined that individual creativity served as a mediator between social norms and leadership. Li and Wang investigated the impact of paternalistic leadership on innovation. They then proposed a theoretical model that used the three dimensions of paternalistic leadership (i.e., benevolence, morality, and authoritarianism) as the dependent variables, constructive deviance as the mediating variable, and innovation performance as the dependent variable. The empirical results showed that benevolent and moral leadership had a positive impact on innovation performance, while the impact of authoritarian leadership was negative. Benevolent and moral leadership had a positive impact on innovation performance through the mediating effect of constructive deviance, whereas authoritarian leadership had a negative impact.

Liu X. et al. explored the mechanisms behind the influence of specialized knowledge search on sustaining and disruptive innovation. They found that sustaining innovation was positively associated with knowledge search strategies, while disruptive innovation had an inverted U-shaped correlation with the supply chain and market knowledge search and had a positive correlation with scientific knowledge search. Liu conducted research that aimed to improve the effectiveness of knowledge-based talent management on technology enterprises to promote development stability. The findings indicate that knowledge workers were generally satisfied with companies' organization and management, but dissatisfied with the training and promotion systems.

Liang et al. conducted a survey among 215 executives. They determined that both entrepreneurs' psychological capital and entrepreneurial opportunities had a significant positive correlation with financial risk expectations. Using both virtual reality scenarios and experimental methods, Hu and Zhang explored how the implantation of an organizational culture of innovation and entrepreneurship affected people's psychological cognition in the process of spatial narrative. Experiments have confirmed that implanting an entrepreneurial organizational culture positively affects people's psychological cognition in spatial narrative. Workspaces combining design innovation and entrepreneurial organizational culture help inspire employees' senses of belonging, collectivity, pride, purpose, and joy at work. Yang and Wu investigated the correlation between work environment, employee innovation, and firm performance. The findings demonstrated that both work environment and employee innovation promoted firm performance. Additionally, work environment affected the relationship between employee innovation and firm performance. Zhao and Ba explored three key aspects (i.e., data sources, collection strategies, and analytical perspectives) of mobilizing visual methods for studying entrepreneurship-as-practice. This essay bears implications for researchers and educators working at the intersection of entrepreneurship research, the practice theory, and visual methods.

Wang and Deng used secondary data to examine the impact of innovation of executive incentive systems on the cost of equity. They discovered that innovation of executive incentive systems had a positive governance effect on the cost of equity. Particularly, executive compensation incentives significantly reduced the cost of equity. Li J. et al. conducted a comparative analysis of regional and cultural differences of modern Chinese business cliques, as well as differences in entrepreneurs' psychological characteristics and entrepreneurial behavior. They revealed that regional culture had a significant impact on the development of modern business cliques and on the regional economy.

Shen et al. examined the relationship between entrepreneurship and corporate social responsibility (CSR) under marketization conditions. Their results indicate that, when marketization is introduced into the model, entrepreneurship and marketization had a significant positive impact on CSR. Applying the structure–conduct–performance analytical framework, Liu B. et al. analyzed the normalization of CSR by Chinese startups after the outbreak of the COVID-19 pandemic. They suggested that CSR for startups should not be limited to altruism, but should include altruistic and self-interested mechanisms.

## New Entrepreneurs

Within the Chinese cultural context, Chen Y. et al. explored the influence of error management on entrepreneurial self-efficacy and innovation under the perspective of Zhongyong thinking (Doctrine of the Mean). Results indicate that the Zhongyong thinking played a moderating role in how error management influences innovation behaviors. Han analyzed the impact of venture capital and investment strategies on the innovation performance of Chinese startups from multiple perspectives. The author found that, unlike private and foreign venture capital, national venture capital significantly improved innovation performance. Through policy content analysis and questionnaire surveys, Yu and Du explored the impact of regional competitive advantage, educational psychology, innovation and entrepreneurship policy, and entrepreneurs' innovation consciousness. They discovered that the psychological strength of technology-based entrepreneurs did not meet the required standard, and their innovation consciousness was lacking. Hence, they were unable to fully utilize the regional competitive advantage to resolve problems that require innovative entrepreneurship.

Lin et al. examined when and why opportunity novelty promotes (inhibits) opportunity adoption. They discovered that entrepreneurs with a low-level construal mindset tended to perceive more risk in novel opportunities, reducing the likelihood of opportunity adoption. However, novel opportunities have a positive effect on entrepreneurs' perceptions of creativity, increasing the likelihood of opportunity adoption. Yu W. et al. explored how different types of entrepreneurial networks and decision-making affected the identification of entrepreneurial opportunities. The results revealed that a non-linear decision-making process played a full mediating role between heterogeneous networks and innovation opportunities, while linear decision-making played a partial mediating role between homogeneous networks and imitative opportunities. Shi and Wang analyzed the motivations of new entrepreneurs from the perspective of transitional economy. They found a significant correlation between the survival of young ventures, the motivation to seize opportunities, and subjective and social psychology.

Ye and Dong conducted a questionnaire survey among entrepreneurs of 100 multinational ventures. They aimed to investigate the impact of cross-cultural adaptation on entrepreneurial psychology and intentions. They found that experience (in terms of years) affected cross-cultural competence, adaptability, and entrepreneurial intentions. However, it had no effect on psychological adaptation. Chu et al. used qualitative comparisons to explore entrepreneurial environment and entrepreneur psychology for startups. They found that entrepreneurial psychology, market environment, and entrepreneurial policies were core conditions to improve entrepreneurial effectiveness.

Chen and Tao analyzed the relationship between entrepreneurs' psychological capital and performance. They found that, in addition to optimism, the three other sub-dimensions of psychological capital (i.e., self-efficacy, wish, and toughness) had a positive impact on the performance of new ventures. Li S. et al. investigated the combined impact of entrepreneurial optimism and labor law on the performance of new ventures. They discovered that each dimension of entrepreneurial optimism had a significant positive impact on business performance, while labor law played a mediating role in the relationship. Using entrepreneurs from small and medium enterprises (SMEs), Zhao et al. explored the relationship between entrepreneurial enthusiasm and performance. The results indicate that entrepreneurial enthusiasm and risk-taking behavior affected performance through work engagement. Yang H. et al. conducted content analysis on the interaction between entrepreneurs and venture capitalists in 79 projects from a venture capital reality TV show. They found that passion and readiness had a significant positive impact on venture capitalists' willingness to invest. Based on entrepreneurial metacognition and emotional complexity theories, Chen and Xu surveyed 218 Chinese SME entrepreneurs. They found that entrepreneurs with more emotional complexity were more likely to use both causal and effectual logic.

By applying backpropagation to a neural network-based time series model, Yu and Zhang analyzed the supply-demand ratio of talent in enterprises and the state of entrepreneurship in the development of new ventures. Their findings revealed a mismatch between the cultivation of and demand for innovative talent. Furthermore, the mental health of young entrepreneurs in Shanghai was significantly worse than that of the national average of China. Busy work schedules and multiple societal goals were the main sources of entrepreneurs' stress, increasing the likelihood of severe mental health problems. Fan applied industrial cluster theory and multi-structural models to analyze the factors that influence the cultivation of new entrepreneurs. The results indicate that age and psychological characteristics were key factors that affected entrepreneurial performance and, thus, serve as a basis for cultivating diverse entrepreneurial talent. Su and Li applied the technology acceptance model to investigate new entrepreneurs that participate in online entrepreneurship education. They found that perceived usefulness, ease of use, and trustworthiness had a positive effect on new entrepreneurs' intentions to participate in the training.

Ma et al. adopt meta-analysis to analyze the literature to understand the role of experience and gender in entrepreneurs' business planning activities. Moreover, they found that both management experience and entrepreneurial experience positively influence the entrepreneurs' subsequent business planning behaviors, which contradicts some existing beliefs that inexperienced entrepreneurs are more likely to develop business plans. Li Y. et al. examined the influence of establishing city brand image on the psychological capital of new entrepreneurs. Their results suggested that, in the process of shaping city brand image, enhancement of urban culture is beneficial for emerging enterprises, and cities with more comprehensive urban culture were more attractive for highly educated entrepreneurs.

Based on a survey among 42 entrepreneurial teams in China, Hou et al. examined the relationship between knowledge diversity and team creativity. They found that knowledge sharing and member creativity played a mediating role in the relationship between knowledge diversity and team creativity. Using data from 419 academic entrepreneurial teams, Ye S. et al. explored the mechanisms behind the impact of entrepreneurial teams' expertise heterogeneity on decision-making. They found that team knowledge integration played a mediating role in the relationship between expertise heterogeneity and decision-making, while team reflexivity played a mediating role between expertise heterogeneity and team knowledge integration.

## Education and Curriculum

Using grounded theory, Yu and Jiang surveyed several leaders of the digital media art design course and entrepreneurs who achieved notable success in the digital media art design after graduating. They established a theoretical model outlining a reform path for innovation and entrepreneurship education in digital media art design. An and Xu studied how the virtual entrepreneurial practice affected the “cultivation of university entrepreneurial talents and innovation and entrepreneurship education.” They found that the respondents have not formed independent entrepreneurship concepts. Moreover, their basic entrepreneurial quality is relatively poor, their entrepreneurial awareness is weak, and their entrepreneurial psychological quality is relatively poor. Using literature analysis and questionnaire survey, Chen Z. et al. investigated contemporary college students' entrepreneurial value judgments. They proposed strategies for constructing college students' entrepreneurial value judgments, including environment, system, method, and mechanism construction. Using a case study method, Wang X. et al. examined the factors affecting the formation and operation of a university entrepreneurship ecosystem. Their results revealed that extracurricular activities, networking, entrepreneurial culture, and leadership significantly affect the formation and operation of a university entrepreneurship ecosystem.

Wang Z. et al. used experimental methods to explore entrepreneurship teaching methods (e.g., traditional teaching, case study, and scene simulation method) and study the impacts of entrepreneurial experience and ability on students' entrepreneurial psychology. Moreover, they showed that the scene simulation method significantly impacts both entrepreneurial psychology and ability in students. Combining both quantitative and qualitative research, Cheng et al. explored the potential factors affecting student satisfaction in flipped teaching. They found that “collaborative learning and cognitive demands” are important predictors of learning satisfaction. Yang Q. et al. divided entrepreneurship educational methods into classroom teaching and extracurricular activity methods from the ability level training perspective. Their findings indicated that although the direct effects of the two teaching methods are similar, the extracurricular activity method can effectively improve entrepreneurial attitudes and perceptions of behavioral control, positively affecting entrepreneurial intentions to a greater extent.

From the educational psychology perspective, Liu and Yu analyzed the awareness of college student startup entrepreneurs toward mass entrepreneurship and innovation. Their results showed that nearly 20% of the students have a comprehensive understanding of innovation and entrepreneurship and that <10% of the students are interested in innovation and entrepreneurship activities. Additionally, the vast majority of students believe that entrepreneurship education is a single and simple curriculum that is irrelevant to their majors. In the innovation and entrepreneurship context, Gao et al. explored the factors influencing students' satisfaction with the teaching quality of the basic entrepreneurship course. Their results showed that teaching contents, methods, conditions, and management are significantly and positively correlated with the teaching quality of an entrepreneurship course and are important factors affecting students' satisfaction. Hu W. et al. conducted research and formulated specific solutions from the aspects of teaching objectives, contents, methods, and models and course performance assessments. Moreover, they made theoretical and practical contributions to cultivating innovative and entrepreneurial abilities of design talents.

Interest in intrapreneurship research is growing, but the contributions to this field remain fragmented, and the field lacks a comprehensive framework. Huang et al. proposed a conceptual framework including both enabling factors (at the individual and organizational levels) and facilitation mechanisms, upon which enterprises can cultivate intrapreneurship capabilities. From an intrapreneurship perspective, Rose et al. explored the antecedents of shared leadership in innovation labs and its impact on team creativity. Based on existing research on shared leadership and innovation, they proposed the antecedents of innovation teams, namely, experimental culture, task reflexivity, and voice.

## Talent Cultivation

From the perspective of the theory of reasoned action, Chen S. et al. conducted a survey among graduating students from their undergraduate degrees. They found that attractiveness of the local environment and family characteristics were the most important factors that affect students' choice between entrepreneurship and seeking employment. Family characteristics have a significant positive impact on their attitudes toward entrepreneurship and employment, subjective norms, and willingness to seek entrepreneurship and employment, similar to the attractiveness of the local environment. Based on cognitive behavior theory, Li et al. discovered that government subsidies had a positive moderating effect on exploratory innovation, innovation development, and entrepreneurial intentions.

From the perspective of indirect entrepreneurial self-efficacy, Jiatong et al. explored the direct effects of entrepreneurs' educational background, mindset, and creativity on entrepreneurial intentions. The researchers determined that entrepreneurial self-efficacy played a partial mediating role between entrepreneurs' educational background, mentality and creativity, and intentions. Wang W. et al. examined the impact of the COVID-19 pandemic on green entrepreneurial intentions of business school students from the perspective of green entrepreneurial self-efficacy, optimism, ecological values, and social responsibility. The findings showed that COVID-19 had a strong beneficial effect on green entrepreneurial self-efficacy, optimism, ecological values, and social responsibility. Based on communication and social cognition theories, Sun et al. analyzed the relationship between entrepreneurial behavior, innovation mode, and entrepreneurial self-efficacy. They found that entrepreneurial self-efficacy played a mediating role between business model innovation and entrepreneurial behavior.

Through a questionnaire survey, Xiao and Wang investigated the current climate of entrepreneurship education for overseas returnees and college students in China. The results indicate that entrepreneurship education received by returnees was more advanced than that of domestic students. College students with overseas study experience demonstrated greater adaptability and had a more optimistic attitude despite difficulties and setbacks. Qi et al. conducted a questionnaire survey and examined the entrepreneurial status (entrepreneurial motivation and barriers) of design students. It was found that a lack of experience and financial resources were the main barriers, while access to personal wealth and self-worth were the main motivations. Chen and Tu adopted an experimental method to investigate the learning motivations and performance of 600 secondary school students in a digital game-based setting with a competitive structure. The findings indicated that entrepreneurs preferred to be more aggressive in terms of competitions, had a higher requirement for achievement, and were more likely to foster competition in non-competitive environments.

Zhou et al. explored the entrepreneurial psychology of physical education students in the “Internet Plus” environment. They found that entrepreneurial self-efficacy effectively predicted entrepreneurial intentions. Wang Q. et al. employed a case study to explore psychological pressures encountered by entrepreneurs, the psychological capital of college athletes, and the cultivation of employability. The results indicate that career guidance courses had a significant positive effect on students' employability, whereas psychological capital played a mediating role in this relationship. Additionally, the psychological stress of entrepreneurs had a positive impact on entrepreneurial performance.

Wang and Jiang examined the impact of entrepreneurship on college students' entrepreneurial abilities and entrepreneurial values in the new media environment. The findings reveal that successful entrepreneurship models could encourage students to receive entrepreneurial education. The respondents reported that they lacked financial support, business skills, capabilities, and understanding of corresponding policies and laws. Zhang J. et al. explored the relationship between new media innovation concepts, entrepreneurship education, and talent that specialized in dynamic expression of creativity. The experimental results revealed that, after training, students' ability to develop innovative concepts and to undertake entrepreneurial self-education through the dynamic expression of creativity, consciousness, and perception was significantly improved. Zhang and Song found that students are keen about entrepreneurial activities. However, 53% of the students have not conducted entrepreneurial activities, indicating that students' entrepreneurial ability is insufficient. Long et al. used the ridge regression model to study the drivers of entrepreneurship education performance of medical students. They found that entrepreneurial courses, teachers, competitions, practices, and policies have significant effects on the entrepreneurship education performance of medical students.

Geng et al. analyzed the current situation of entrepreneurial awareness of college student startup entrepreneurs and explored the role of innovative and entrepreneurial talents in social and economic development. They found that the relative freedom of time and space are the main factors encouraging college students to start their own businesses, while lack of funds and experience are the main constraints to entrepreneurship. Using general systems theory and fuzzy-set qualitative comparative analysis, Jiang et al. examined whether entrepreneurship education enables graduates to start their own companies more efficiently. They demonstrated that combining co-creation type, competition-oriented environment, and entrepreneurship education can produce efficient entrepreneurial activities. Based on the new entrepreneurial psychology and cross-cultural adaptation, Zhou Q. explored the entrepreneurial psychology of college students. They found that achievement motivation has significant impact on entrepreneurial intentions and knowledge levels. By avoiding blind entrepreneurship, college students can better adapt to cross-cultural environments. Under new media art, teachers should optimize the teaching model of the cultivation of innovative and entrepreneurial talents in art colleges and cultivate students' innovative ability and entrepreneurial positive psychology.

This Research Topic aims to understand startups from idea to market from a psychological perspective. The Research Topic comprises 61 research papers from various fields that clearly help us understand the psychology and education of startup development. Finally, we conclude this editorial by thanking all the authors for their contributions to this Research Topic, and all reviewers for their voluntary contributions in the peer review process to maintain the high quality of this Research Topic.

## Author Contributions

YJW conceived of the idea and coordinated the Research Topic. C-HY and M-YC carried out support tasks for the coordination of the Research Topic and Edition of Articles. All authors contributed to the article and approved the submitted version.

## Funding

This work was partially supported by Ministry of Science & Technology, Taiwan (109-2511-H-003-049-MY3), Guangdong Planning Office of Philosophy and Social Science (GD18XJY01), and Guangdong Province Education Science 13th Five-Year Planning Project (2020GXJK420).

## Conflict of Interest

The authors declare that the research was conducted in the absence of any commercial or financial relationships that could be construed as a potential conflict of interest.

## Publisher's Note

All claims expressed in this article are solely those of the authors and do not necessarily represent those of their affiliated organizations, or those of the publisher, the editors and the reviewers. Any product that may be evaluated in this article, or claim that may be made by its manufacturer, is not guaranteed or endorsed by the publisher.

